# PB.28. Determination of recall rates for assessment in women undergoing annual surveillance breast MRI: is a rate <10% achievable?

**DOI:** 10.1186/bcr3720

**Published:** 2014-11-03

**Authors:** N Healy, S Cosgrove, P Wen, T Kwa, F O'Driscoll, G Offiah, Y Roden, S Sebaoui, K Wolohan, S O'Keeffe

**Affiliations:** 1 St James Hospital, Dublin, Ireland

## Introduction

High recall rates from surveillance breast MRI are associated with patient anxiety and increased workload. NHSBSP guidelines recommend a maximum of a 10% recall rate with an expected rate of <7% in high-risk women undergoing surveillance with breast MRI. Our aims were to review surveillance breast MRIs performed at our institution from 2009 to 2013 to determine the recall rate and cancer detection rates.

## Methods

Surveillance MRIs performed in women at high risk of developing breast cancer over a 5-year period were reviewed. Breast MRI was performed using a standard protocol on a 1.5 Tesla MRI. For all patients with a BIRADS MRI 0, 3, 4 or 5 score, additional imaging, modality of biopsy and histology were recorded.

## Results

A total of 1,119 surveillance breast MRIs were performed over the 5-year period. These included women with a known genetic mutation or those at high risk based on genetic assessment. In total, 121 (10.8%) had BIRADS MRI scores which required recall for further imaging. Seventy-one patients (58.6%) had a biopsy performed and 19 cancers were detected, giving an overall cancer detection rate of 1.7%. Eleven (9%) were invasive ductal tumours. Of those recalled, 74 (61%) were in the prevalent round of screening.

## Conclusion

While our data compare favourably with published data, the breast MRI recall rate is greater than the expected 7% recommended in the UK. This may be due to large numbers in the prevalent round.

